# Bees as Biosensors: Chemosensory Ability, Honey Bee Monitoring Systems, and Emergent Sensor Technologies Derived from the Pollinator Syndrome

**DOI:** 10.3390/bios5040678

**Published:** 2015-10-30

**Authors:** Jerry J. Bromenshenk, Colin B. Henderson, Robert A. Seccomb, Phillip M. Welch, Scott E. Debnam, David R. Firth

**Affiliations:** 1 Bee Alert Technology, Inc., 91 Campus Drive, PMB# 2604, Missoula, MT 59801, USA; E-Mails: bobseccomb@gmail.com (R.A.S.); phwelch@mindspring.com (P.M.W.); 2 Division of Biological Sciences, University of Montana, 32 Campus Drive, Missoula, MT 59812, USA; E-Mail: scott.debnam@umontana.edu; 3 Missoula College, University of Montana, 32 Campus Drive, Missoula, MT 59812, USA; E-Mail: colin.henderson@umontana.edu; 4 School of Business Administration, University of Montana, 32 Campus Drive, Missoula, MT 59812, USA; E-Mail: david.firth@umontana.edu

**Keywords:** honey bees, biosensors, proboscis extension reflex, scent, bee counters, scale hives, infra-red imaging, LIDAR, RFID

## Abstract

This review focuses on critical milestones in the development path for the use of bees, mainly honey bees and bumble bees, as sentinels and biosensors. These keystone species comprise the most abundant pollinators of agro-ecosystems. Pollinating 70%–80% of flowering terrestrial plants, bees and other insects propel the reproduction and survival of plants and themselves, as well as improve the quantity and quality of seeds, nuts, and fruits that feed birds, wildlife, and us. Flowers provide insects with energy, nutrients, and shelter, while pollinators are essential to global ecosystem productivity and stability. A rich and diverse milieu of chemical signals establishes and maintains this intimate partnership. Observations of bee odor search behavior extend back to Aristotle. In the past two decades great strides have been made in methods and instrumentation for the study and exploitation of bee search behavior and for examining intra-organismal chemical communication signals. In particular, bees can be trained to search for and localize sources for a variety of chemicals, which when coupled with emerging tracking and mapping technologies create novel potential for research, as well as bee and crop management.

## 1. Introduction

Observers of honey bee behavior as early as Aristotle [1] down through Butler [2] and Lineburg [3] noted, as colorfully described by Butler in 1609, that flying bees perceive scents below them, and “presently (take wind of it)” from sources “afar off”. The long history acknowledging the importance of chemical cues notwithstanding, in the 1940s, the discovery of a bee dance language ushered in a new paradigm.

### Scent-Directed Search is Under Valued by Focus on Dance Language

First described by von Frisch in 1937 [4,5,6,7], evidence that an insect society may have a symbolic language that communicates directions and distances of nectar and pollen resources among foragers swept the scientific world by storm [8,9,10]. Von Frisch refined his dance language hypothesis [5] over nearly a decade of research and was awarded a Nobel Prize in 1973. The dance language paradigm for honey bee foraging behavior holds forth, virtually unchallenged, to this day.

As an unassailable paradigm, the perception that bee foraging is a socially coordinated behavior inhibited serious exploration of the chemosensory abilities of bees. In the 1960s, Wenner, a professor of natural history and later Provost of the College of Creative Studies at the University of California, Santa Barbara, focused his doctoral and subsequent research on an expansion of von Frisch’s work. He soon discovered that his predecessors had failed to observe that “bees learn quickly (the conditioned response phenomenon, as with Pavlov and his salivating dogs)” to respond to chemical cues [8]. He also concluded that honey bee recruitment success could be explained solely by their reliance on scent, a hypothesis first proposed and then rejected by von Frisch in 1939 [10]. Wenner’s hypothesis was met with such strong opposition by dance language advocates that he was forced to re-direct his research away from bees. He finally returned to bee olfaction research two decades later, incidentally publishing treatises on how science advances in general [11].

Controverting the common misconception that bees have a poor sense of smell that is not much more refined than that of humans, honey bees have a superb ability to detect chemical signals. In laboratory and field tests, they have proven able to recognize a vast array of different compounds at parts per trillion, even parts per quadrillion, vapor concentrations [12]. Johnson and Wenner [9,13] showed definitively that bees could be conditioned to search for odors by providing them with a rich syrup reward. This led to our own research into methods and technologies for using bees as living biosensors for a wide variety of chemical signals [14,15,16]. We also found that like dogs and other vertebrates, invertebrates such as bees learn to associate a particular odor with a reward. Compared to dogs which usually take months to train, honey bees can be conditioned or trained to respond to one or several volatile chemical compounds in less than an hour in the laboratory. Whole colonies, 15 or more at a time, can be conditioned in less than three days in open field settings. In addition, bees tend to be less easily distracted than dogs and do not require the direct guidance of a handler. Recognizing bees’ exquisite chemosensory ability and capitalizing on these behavioral advantages has stimulated development of a number of new approaches relying on bees as a biosensor system that holds great promise.

## 2. Bees as Chemical Biosensors in the Laboratory and in the Field

Bees may be used as chemical biosensors in two ways: (1) constrained in a laboratory-based, benchtop assay termed the Proboscis Extension Reflex system (PERs); or (2) as traceable, free-flying biosensors in the field.

### 2.1. Chemical Signals and Proboscis Extension Reflex

In 1953, Ribbands published two books on the behavior and scent language of honey bees [17,18]. He did not reject the dance language, but he too demonstrated that some of von Frisch’s experiments and results were erroneous. He devoted an entire chapter of his first book to the topic of directing honey bees to crops through scent-training.

He also highlighted research performed in the 1930s by several Russian agricultural scientists [19,20,21,22,23,24,25] which unfortunately was tainted by association with Lysenkoism and that failed paradigm’s insistence that environmentally induced traits were heritable [26,27]. Consequently, his contradictions of dance language and his scent-directed alternative to the dance language paradigm were largely ignored [17,18]. We suspect this was due both to the conflict between contrasting bee-search paradigms and his inclusion of Russian research in support of his own hypothesis. Today, Ribbands’ work is mostly forgotten, but in hindsight, is extremely valuable. He laid out methods for conditioning bees and proposed potential uses of bees as biosensors which are only now being fully appreciated.

He (1953) [17] also explained the origin of the mistaken notion that bees have a poor sense of smell which is not much better than that of humans. Von Frisch (1919) [28] measured the bee threshold of perception for the scents of two pure chemical compounds: bromstyrol and methyl heptenone dissolved in liquid paraffin. Von Frisch then compared his bee perception results for these two chemicals with the perception thresholds of his wife and himself.

Ribbands [17] concluded that the von Frischs’ thresholds of perception for these two scents were not representative of the most sensitive 25% of the human population. Whereas von Frisch concluded that bees could only recognize methyl heptenone in a 1:2000 dilution, he found that bees perceived the chemical at 1:40,000,000 dilution. Ultimately, Ribbands [18] found that bees performed even better in their perception of many pheromones and floral scents.

In his chapter on bee taste and smell [17], Ribbands provided a detailed history of development of the Proboscis Extension Response (PER) assay to investigate bee perception of chemicals. Dὅnhoff (1855) [29] observed that honey bees stretch out their tongues when their antennae are touched with a glass rod dipped in sugar solution or honey, but not if the rod was dipped in water. By 1935, Marshall had developed a simple version of the modern PER system (PERs) [30] whereby live bees are constrained in a holder and conditioned to respond to chemicals using a sucrose syrup reward.

### 2.2. Constrained Bees as Chemical Biosensors

In about 2000, Inscentinel, Ltd., a United Kingdom-based biotechnology company, began specializing in harnessing the olfactory ability of insects for trace vapor detection with *parts per trillion* sensitivity [31]. Based on an adaptation of the PER assay methodology described briefly above, the company developed a bench-top, environmentally stable cassette and bee-holder, using a camera and image processing to monitor the PER behavior of sets of three bees. Working with another company, Panchromos, Ltd., a hand-held, battery-powered, bee-holder and detector for field testing was later produced. Named the Inscentinel VASOR (Volatile Analysis by Specific Odour Recognition), the device held 36 bees, could be switched from filtered to unfiltered air intake, and used a step-wise change in odor concentration to elicit PER. In both the bench-top and field-portable devices, sets of bees were used as the specific odor recognition biosensors. Under the US Defense Advanced Research Projects Agency’s (DARPA) Controlled Biological and Biomimetics Systems Program, Inscentinel scientists came to work in the USA; first with us at the University of Montana (UM), and later with researchers at the US Department of Energy’s Los Alamos National Laboratory.

Inscentinel’s goal was to commercialize PERs for civilian and defense-related monitoring. Their systems used environmentally controlled containers fitted with bees trained to specific compounds. Bee PER response was derived from data obtained by video processing systems. Despite demonstrated technical feasibility of the system, logistical constraints appear to have stalled the acceptance of Inscentinel’s PERs detector. However, interest in PERs by other investigators remains high, with a renewed focus on standardizing procedures [32,33].

Our own use of PERs has been mainly for screening bees’ ability to detect chemicals of military concern. We have also assisted researchers in plant crop genetics and pollination in the US and New Zealand. These biologists use PERs to screen plant varieties for attractiveness to bees and to identify the chemicals to which bees are attracted. From collaborations with a US seed company and now with researchers in New Zealand, we have good evidence that Ribbands and the Russians were correct: bees can be directed to pollinate specific crops using appropriate conditioning methods.

**Figure 1:**
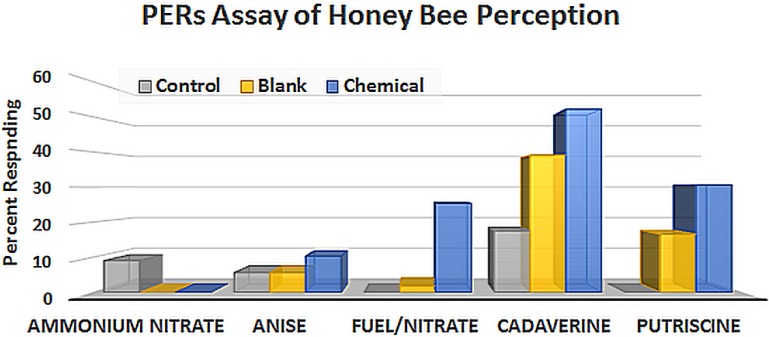
PERs results for chemical detection of fertilizer, fertilizer-based bombs, and decomposition products of animal carcasses compared to anise, a floral scent.

PERs has typically been used to study associative learning in honey bees, especially with respect to floral scents. More recently, the method has been extended to studying the effects of pesticides on learning [34,35], and the following chart (Figure 1) presents PERs results for a sample of novel compounds that can be detected by bees at low concentrations. This data is from a short trial from our military studies whereby chemicals associated with dead bodies (cadaverine, putrescine), fertilizer-based Improvised Explosive Devices (IEDs) (fuel/nitrate), and fertilizer (ammonium nitrate) are compared to anise, a plant attractant to which bees are readily conditioned.

### 2.3. Whole Colonies Used in Environmental Monitoring Studies

Local and landscape-scale biomonitoring has been conducted using *passive detection*, which capitalizes on natural behaviors of organisms. Beginning in 1974 up until 2000, we conducted numerous landscape-level surveys and mapping of pollutant dispersal over thousands of hectares. Forager bees from colonies placed in areas of interest bring back numerous environmental chemicals from air, soil, water, and vegetation that then accumulate in their hives. We published a seminal paper of our methodology in Science in 1985 [36]. Beyond using bees as passive biological monitors, that research pioneered the application of Kriging to biological survey data. Kriging or Gaussian process regression is a method of interpolation for which the interpolated values are modeled by prior covariances. Kriging allowed us to construct chemical-specific, risk-exposure isopleth maps [36].

Other investigators, especially in Europe, also began using bees for bioenvironmental monitoring. A 2003 review of bees and chemical monitoring [37] details numerous examples of applications of honey bees as biomonitors. This type of investigation continues to this day. Recent uses of bees as passive detectors or monitors include periodic analysis of honey collected by bees near eight airports [38] in Germany and monitoring chemical pollution in the Kurdistan Province of Iran [39].

In contrast to passive detection where bees free roam, *active detection* is directed search. This can be accomplished in a variety of ways, ranging from modifying the natural foraging and search behaviors of an animal to driving the animal by some form of technological interface. For example, DARPA investigated controlling cockroach movement using an electronic neural interface mounted on the back of the insect [40].

DARPA’s exploration of the potential for training insects to find materials of interest to the US military led to our own development of bee-training protocols (operant conditioning). During this decade-long research we tested and validated a large number of chemical and biological agents of interest to the military. Our field-based bee conditioning systems [16] are autonomous devices for: (1) directing thousands of bees to search for unique chemical signals such as those leaking from landmines [15], drug laboratories, dead bodies, or chemical signals produced by diseased plants and animals; (2) focusing bee foraging on specific crops; and (3) chemical signal detection requiring canvassing of large areas.

**Figure 2:**
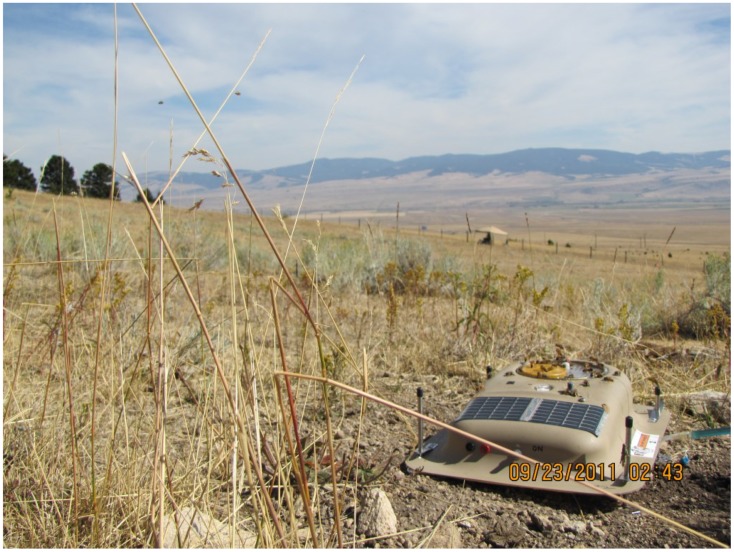
Solar-powered, micro-processor-controlled bee-conditioner system with scent and reward (proprietary syrup solution) dispensing systems. Reward intervals can be controlled automatically by the device, re-programmed as needed, or remotely controlled by wireless communications.

Just as Inscentinel’s instrumentation hardly resembled the straws and index cards initially used to constrainer bees for PERs testing, our solar-powered, micro-processor-controlled, wireless-networked bee-trainers (Figure 2) [16] bear little resemblance to primitive odor-delivery systems used by the Russians in the 1930s. However, the underlying operant-conditioning concepts and methods are similar.

## 3. Technological Advancements in Bee-Based Biosensors/Biomonitors

### 3.1. Bee and Hive Monitoring

**Figure 3:**
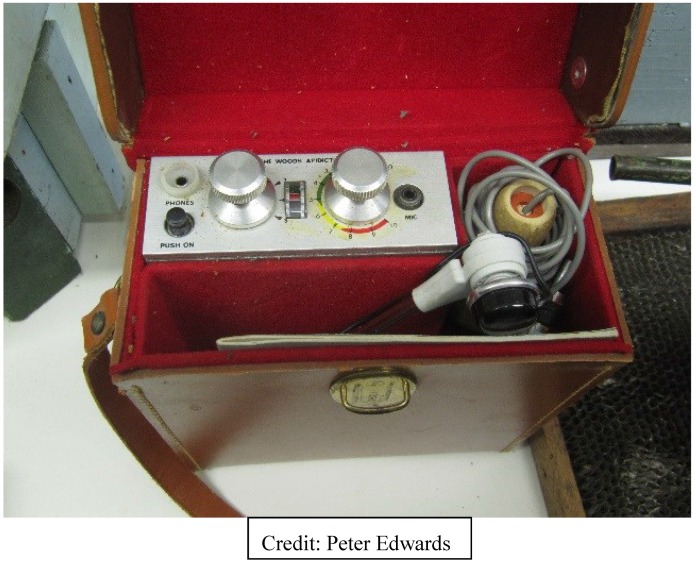
Wood’s Apidictor for honey bee colony swarm detection.

The first marriage of electronic sensors and bees was a frequency analyzer called the Apidictor (Figure 3), developed, patented, and sold during the 1960s by a UK beekeeper and sound engineer, E.F. Woods [41]. The Apidictor was a frequency band-pass filter for detecting sound changes which occur in beehives up to two-to-three weeks before a bee colony swarmed. Work on a modern Apidictor has re-emerged in Europe, and Wood’s schematics are available on the Internet. There also is video of a 2012 iPhone-based Apidictor. In 2011, Bencsik *et al.* [42] described their swarm detector using accelerometers to analyze hive vibrations.

The original Apidictor was followed by two inventions at Oak Ridge National Laboratory in the USA: (1) research-only, prototype tags for marking and tracking bees [43,44]; and (2) an acoustic device for discriminating races of bees [45] based on wing-beat frequency. Simple hive entrance-mounted bee activity/movement counters were mentioned and discussed in the 1980s. The first commercial bee-counters were marketed by a Belgian electronics company in the mid-1990s [46]. In 1995, we deployed the first electronic SmartHives^℗^ (UM, Missoula, MT, USA), equipped with scales, bee-counters, temperature and relative humidity sensors, digital weather stations, and telephone communications for remote monitoring via the Internet [47,48,49].

Beginning in 1995, significant funding from the US Army and DARPA, concurrent with advances in sensor and electronics technology, resulted in a proliferation of bee and pollinator-related sentinel, monitoring, and chemical signal detection methods and technologies. Together, these technologies are posed to radically change bee management, agricultural practices, and research on chemical signals between organisms. As such, a summary history and overview of current and emerging technologies, especially those involving electronics and distance communications of data, is warranted.

### 3.2. Detecting and Tracking Bees Marked with Tags

Major developments in the fields of electronics and bees were initiated in the mid- to late 1980s by a research team at Oak Ridge National Laboratory (ORNL). Howard Kerr, an ORNL engineer and beekeeper, lead an effort to identify and track Africanized bees. First, an acoustic detector to discriminate between European and Africanized bees was developed and patented [45]. This was a hand-held instrument with a clear capsule into which a bee could be released and allowed to fly toward the light of the sun. Frequency filters analyzed wing-beat frequency, and a warning light indicated detection of Africanization. This device discriminated European honey bees from the Africanized variant based on subtle differences in the wing-beat frequency of these two strains of bee. 

Second, his team produced an electronic tracking chip for bees [43] that was glued onto the thorax of the bee. The chip weighed less than 35 mg and was about the size of a half-carat diamond. It achieved its low weight by eliminating batteries, instead using tiny solar collectors for power because batteries would have been too heavy. The transmitter chip had a range of about a mile and used a ground-based sensor to read infrared light pulses from the chip. None of these devices became commercially available. Catching bees to put in the acoustic detector was problematic, reliable discrimination across the full-range of hybridization between the two different strains of bees was difficult, and the tracking chips and readers were too expensive for general use. 

The next major development in tracking bees was by Gerald Loper, Wayne Wolf and Orley Taylor, Jr. of the United States Department of Agriculture’s (USDA) Carl Hayden Bee Research Laboratory in Tucson, Arizona. They studied queen mating using x-band radar to document drone flyways and congregation areas [50,51]. Because drones are larger than worker bees and congregate in large numbers in mating areas, no markers were needed to identify drones in their studies. Radar is best suited to large masses of insects, e.g., swarms of insects, but can detect small insects at close ranges and large insects at longer distances. Without reflective tagging, however, radar cannot track individual insects. 

**Figure 4:**
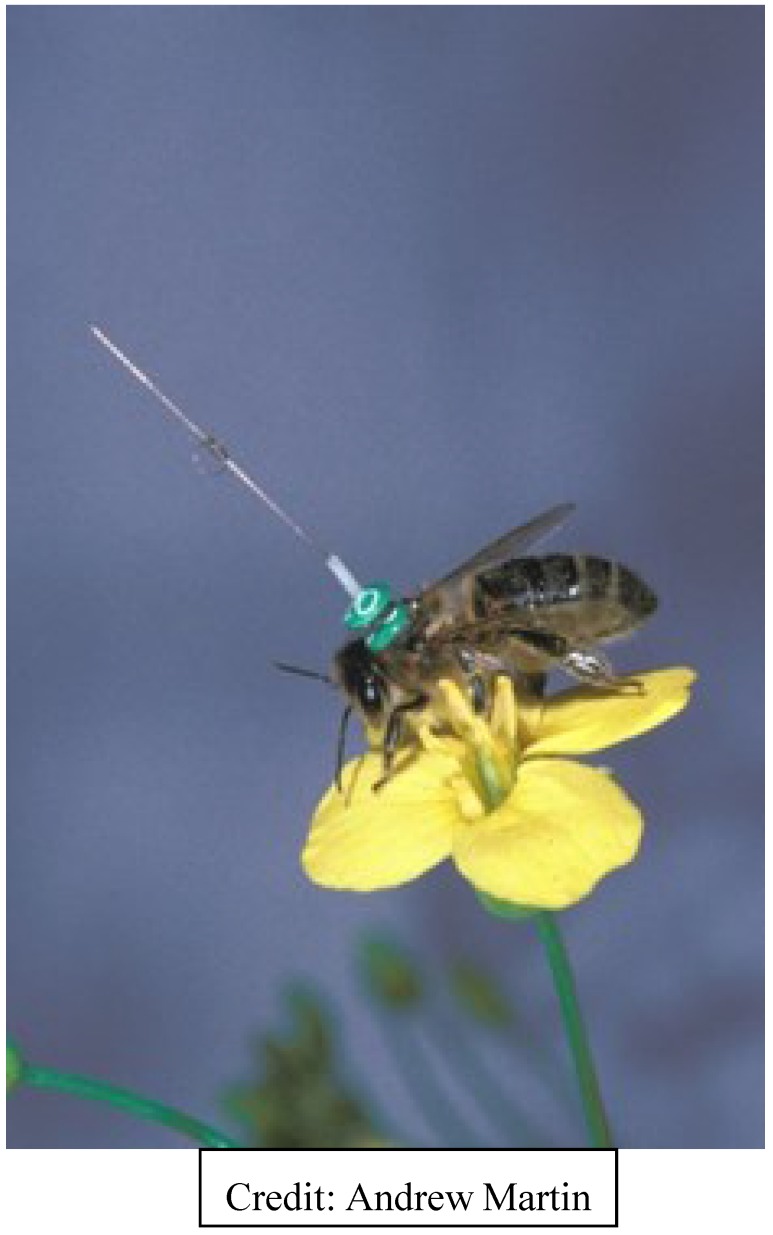
Harmonic radar marking and tracking chip. Note the tall, vertical antennae.

Shortly thereafter, Joseph Riley, at the Natural Resources Institute (NRI) Radar Unit, Worcestershire, UK, and his team of engineers and investigators re-visited the chip marker approach to tracking bees. An idea to track bees came from the Rothamsted Experimental Station; NRI solved the problem [52]. The NRI unit later became part of Rothamsted Research. Riley’s team successfully followed flying bees using hand-held readers and harmonic radar chips fastened to bees (Figure 4) [53,54,55,56,57]. Unlike most other radio-frequency tags, harmonic radar tags re-radiate the radio-frequency (RF) signal at twice the frequency; a 917 MHz signal will be returned at 1845 MHz.

Although harmonic radar was developed at NRI, the idea to track bees came from Rothamsted Experimental Station. The NRI Radar Unit later became part of Rothamsted Research, and remains there. Drawbacks of harmonic radar were and continue to be: (1) the large size of chip-borne antenna required to increase the detection distance for tracking; (2) the difficulty of tracking insects in a cluttered environment; and (3) the inability to identify individuals in a tagged group of insects. The antenna can and often does alter normal flight, although it is less of an issue with bumble bees which are often considerably larger than honey bees. Still, there have been several honey bee-tracking studies published using this technology, and at least two published reviews [52,57]. Some of the studies, discussed at the end of this article, address the dance language. The latest is a 2014 study using harmonic radar to detect the effects of *Nosema* infection on the homing ability of honey bees [58].

Where antenna length is not an issue, harmonic radar is a very useful technology. Harmonic radar systems are commercially available and are routinely used for finding avalanche victims. One of our students used it for tracking salamanders, and large insects like butterflies and some beetles can easily carry a harmonic radar tracking tag. 

DARPA funded the next iteration of even smaller RF chips in late 1999. We also tested miniature radio frequency (RF) pit tags and hive-mounted readers developed for us by a group of engineers directed by Ron Gilbert at Pacific Northwest Laboratory. Each 27 mg RF tag was about half the size of a rice grain and contained a 10-character code which identified an individual bee. When the bee entered or exited the hive, an electronic tag reader recorded the passage. Unfortunately, we concluded that our miniature chip was still large enough to alter normal bee flight and that read range was limited to centimeters [59].

Much progress in RF tags has been made in the past decade. Tiny passive tags using nanoblock technology are now employed for inventory control in warehouses and stores such as Wal-Mart. In fact the tags produced by Alien Technology, a world leader in volume production of Radio-Frequency Identification (RFID) products, were produced by Gilbert’s team—the same team that placed the first RFID tag on a bee for us.

Small and light, passive (2–3 mg) RF bee tags became commercially available around 2006 in Europe. These have been used to study a variety of bee and colony behaviors, queen mating flights, and the effects of neonicotinoid pesticides [34,60,61,62,63,64,65,66,67]. A particularly ambitious study was launched in 2014 in Tasmania, Australia, where 5000 bees were tagged to study bee movements as the tagged bees passed stationary readers [68].

**Figure 5:**
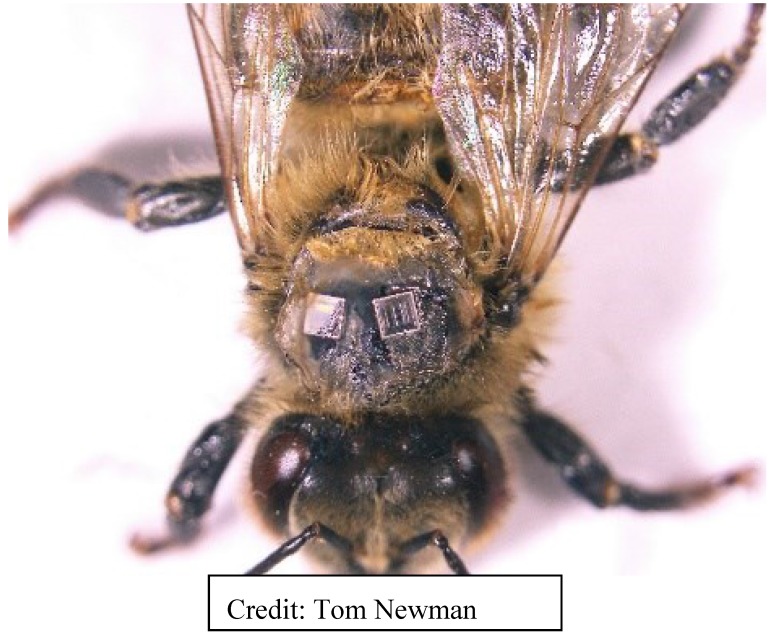
Current size of miniature RFID tags used by investigators [69].

Other investigators [69] mounted even smaller RFID (Figure 5) tags on the thoraxes of bees to study inter-individual variation within a bee colony and plasticity in honey bee foraging activity. Typically, small RFID tags usually have read distances of less than 50 cm; the smallest bee tags have a range of ≤20 mm. To this day, the read range of small passive chips mounted on insects tends to be very limited (in the millimeter to, at most, centimeter range), greatly reducing the detection probability of any marked bee in a free-flying environment. Whether using harmonic radar or RFID tags, the engineering limitation is that detection distance is a function of frequency and antenna size.

Antennas (Figure 4) on tracking chips increase range of detection but add weight and interfere with bee flight behavior. The newest bee tags use an antenna printed on the chip and in one novel study, we experimented with the laser-plating of fractal antennas onto the bees themselves, working with a US Naval Research Laboratory. Active, or powered, chips have greater range than passive tags, but batteries add weight. The solution may be using the insect itself to power the tag.

DARPA continued to focus on developing RFID tracking chips. This resulted in a smaller chip, produced by Mayo Research Institute, followed by efforts to extend the range to one kilometer, which were attempted by at least one large military contractor. To our knowledge, none of these chips have progressed beyond prototypes, despite large financial investments made in developing RFID for long-range location and tracking of insect biosensors. What is clear is that interest in hive- and bee-based sensors, often with advanced, miniaturized electronics has become common enough that there is sufficient information to warrant a book about this and related subjects [60].

### 3.3. Light Detection and Ranging (LIDAR) Tracking and Mapping of Honey Bees

With the short range issues for RFID and the focus on individual bees, and since our primary methods rely on mass conditioning of bees, we re-directed our efforts to developing image subtraction, acoustic detection, and laser for locating, tracking, and mapping bees. Related to our DARPA project, our collaborators at Sandia National Laboratories showed that a LIDAR system could be used to track honey bees, providing both range and coordinates of the bees at a distance of 1 mile (1.6 km) [14]. However, Sandia’s instrument was intended for atmospheric studies and, as such, was large, and the LIDAR beam was not eye-safe for humans who might be down-range from the instrument.

Having established that LIDAR could be used for locating, tracking, and mapping bees, our research team, representing UM, Montana State University (MSU), and Sandia Laboratory, obtained patents and published a series of studies to develop and use both visual monitoring and LIDAR to map bee locations [14,15,16,70,71,72,73]. The majority of the verification research was conducted using an Army test field in Missouri. The instruments used for these studies were mostly research prototypes.

**Figure 6:**
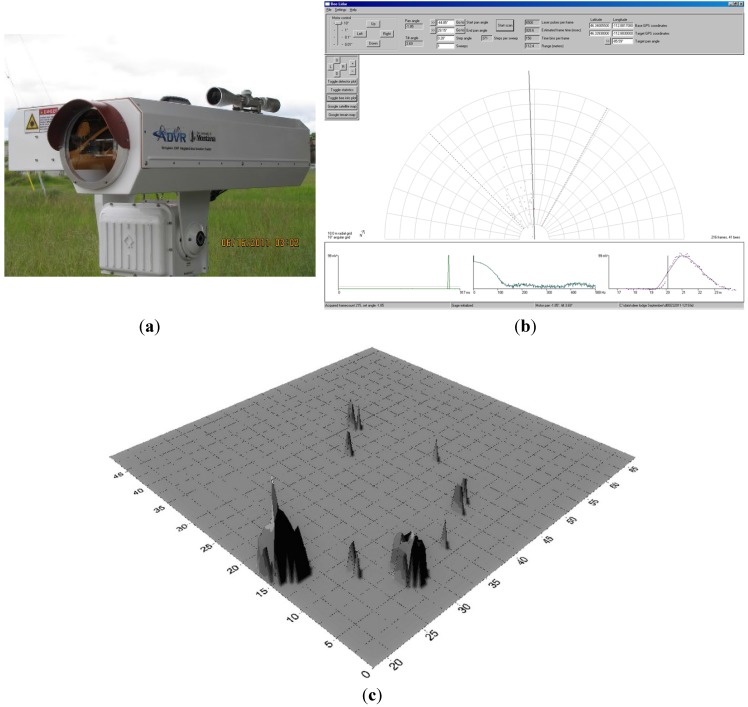
(**a**) Commercial prototype of bee-mapping LIDAR; (**b**) Instrument display screen. The computer screen display provides real-time detection of individual bees (*i.e.*, dots shown in the sweep). Charts at the bottom characterize signal duration, shape, and frequency for each detected insect. Banner at the top gives instrument settings and controls. Most recent bee detection appears on screen in red; (**c**) Three-dimensional density map of actual bee locations across a field from one of our trials using bees conditioned to find a unique scent. The larger and taller the vertical “cone”, the more bees at that spot, and concurrently, the higher the vapor concentration of the subject chemical.

Since 2012 we have been using and testing two commercial prototype bee-mapping LIDAR instruments. These instruments are relatively compact, ruggedized, decimeter resolution LIDARs with built-in image mapping and both 2-D and 3-D visualization capabilities (Figure 6a–c). The LIDAR software includes modules for visualization, contouring, and surface modeling software from Golden Software in Colorado [74]. LIDAR management, display, and visualization software runs under Microsoft Windows.

### 3.4. Electronic Hives, Bee Counters, Chemical Monitoring at the Hive

With honey bees and other social insects, the nest presents added opportunities for bio-sensing. For bees, the hive is the place from which foragers stage numerous forays during the day and to which the entire forager force returns at dusk. From 1973 through the early 1990s [36,37,48,49,75,76,77,78,79,80,81,82], we capitalized on bees sampling surrounding environments, trapping at the hive, and analyzing samples of pollen, nectar and honey, wax, and even the bees themselves. We later found that bees’ electrostatic charge improves their effectiveness as samplers and modeled particle size attraction as a function of charge for both chemical and biological (microbes/viruses) agents [83,84].

In 1995, the US Army’s Center for Environmental Health Research (USACEHR) contracted with us to explore using colonies of bees as rapid response “miners” canaries to both screen military waste sites for toxic chemicals and to provide surveillance of releases of any chemical toxic to bees this is likely to be toxic to people. We put the first electronic surveillance beehives on-line in 1995 [48,49]. Fifty SmartHives^℗^ were used to monitor for releases of toxic chemicals at military toxic waste sites at Aberdeen Proving Ground (APG), just north of the city of Baltimore.

Each hive was set up to permit environmental sampling for volatile (VOCs) and semi-volatile (SVOCs) chemicals. This was done using a combination of commercially available air sampling pumps, carbon molecular sieve tubes, and thermal desorption/gas chromatography/mass spectrometry (TD/GC/MS) to screen beehive atmospheres for VOCs and SVOCs [37]. We expected to see combinations of chemicals released by the bees themselves, their forage resources, and hive walls. We did not anticipate the full spectrum of more than 200 VOCs and SVOCs chemicals that we discovered [37], and noted that “What most bee researchers have previously missed is the significant and often dominant presence of airborne contaminants from non-bee sources.” For example, high perchloroethylene (PCE) levels in hives at APG were associated with queen loss from 50% of the colonies’ place near a hazardous landfill.

In 2005, we received the first US patent for an electronic hive [49] fitted with a variety of sensors. We were not the first to place nor to patent electronics on a beehive. Those milestones were achieved by Struye and his associates, who published a design for a bi-directional bee counter, laying the groundwork for Lowland Electronics in Belgium and their line of Beescan, Apiscan and Bumblescan bi-directional bee counters. These counters were made commercially available in the mid-1990s [46]. The pioneering use of the Lowland Electronics counters was for studies of pesticide effects on bee colonies [46]. The Lowland Electronics design counts bees as they pass through portals fitted with infrared emitters and detectors. A bee is counted if it blocks an infra-red (IR) beam.

We built communications capability missing from the Lowland Electronics counter into our SmartHives^℗^. We designed our own bi-directional, infrared bee counters and added to that our custom signal processing hardware and software which reduces the number of false positive data points resulting from bees starting to enter a counter portal, then backing out.

**Figure 7:**
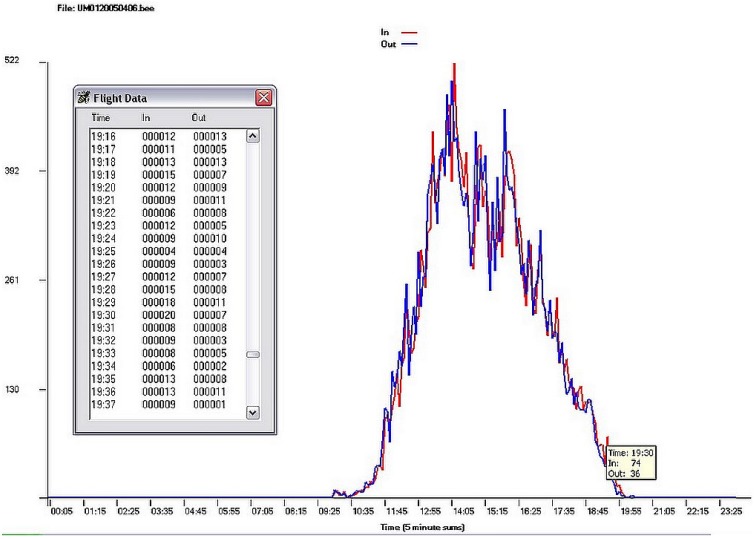
Screen captures of bee foraging data from bi-directional bee counters, 1995. The scrollable table and chart show a single colony’s daily forager activity (bee egress in red, bee ingress out blue) over 24 h. The data table can be accessed by tapping the activity plot at any point. A toggle brings up daily summaries for each colony by week.

These hand-built bee-counting units tend to be expensive and not well-suited to everyday colony management, although the cost has been warranted for some critical research and monitoring projects. We have experimented with alternative technologies for IR-based counters including: capacitance-based counters, visual and infrared image subtraction, still and video imagery (DARPA initiated projects, all documented in our Annual Technical Reports, 1999–2002 [82]). We found IR-based counter systems to be more robust and accurate than capacitance and video-based systems, but IR counters require regular cleaning and maintenance. Our counters have a self-diagnostic program that allows the user to check whether all of the emitters and detectors are working and that none of the passageways are blocked by debris or bees. We developed data summary applications for Windows computers in 1995, later adding touch-sensitive tablets and cell phones (Figure 7).

We learned that users should be cautious about using counters with only a few portals. A properly designed bee counter must have many ingress/egress tunnels or else there will be congestion at the hive entrance, slowing down or blocking bees. In the worst case, this can lead to colony heat prostration. Finally, we learned that the daily return rate for healthy colonies tends to be in the mid- to high 90%. When exposed to an acutely toxic incident, that number can drop to the low 80s or lower. Chronic losses tended to be characterized by a decrease of one to a few percent fewer bees returning each day. We also found that the variability in flight activity from hive to hive at a given location tended to increase with exposure to toxins, as some colonies were affected more than others [48,85].

Recent developments show progress towards accurate and more affordable bee activity counters. A capacitance-based counter (CBC) for bumble bees was described by Campbell *et al.*, and a patent on their capacitance-based counter was obtained [85]. This team and others soon added image-based counting systems, as well as automated systems for monitoring locomotor activity [85,86,87,88,89]. Video activity monitoring is now offered commercially as an option for electronic hives fitted with scales by Arnia Remote Hive Monitoring in Europe and HiveMind in New Zealand. We also have experimented with a capacitance-based counter for our SmartHives^®^. Our preliminary data indicates that queens, drones, and workers all produce a different change in counter capacitance. As such, a CBC should be capable of discriminating movements of drones (males), workers (female), and queens. Campbell *et al.* [85] reported similar findings for their Bumble Bee counter.

We are currently testing our next generation of SmartHives^℗^ in commercial beekeeping operations in the US. These electronic hive systems start with a scale integrated into a wooden or plastic pallet holding four or six colonies of bees. This palletized form factor is commonly used by large-scale, migratory beekeepers in the US. They load, ship, and unload palletized hives on trucks for transport from coast to coast.

These customizable pallet-based systems have a programmable electronic processor, can be powered by alternating current (AC), battery, or a small solar panel, and have options to plug in a wide variety of low voltage analog and digital sensors. The sensor options include but are not limited to: scales, temperature probes, microphones, infrared sensors, relative humidity probes, moisture and fire detectors, movement detectors, counters, and weather stations. These SmartHive^℗^ pallets include on-board data storage. Data retrieval can be via USB port, Bluetooth^℗^, or other wireless communication in the bee-yard. Cellular or satellite communications are used for long-distance communications; they deliver alarms and data to a beekeeper’s desktop computer or cell phone.

Until very recently, limitations in terms of expense, power requirements, processing power, and durability of the necessary electronic components restricted the use of electronic bee and hive monitoring sensors to basic and applied research applications. However, advances within the past five years in essential enabling technologies, such as inexpensive, credit-card-sized computer processors with low power draw, eliminated many hardware limitations. One example is the Raspberry Pi^®^ introduced in 2011 [90]. This is a credit-card sized, Acorn RISC Machine (ARM)-based computer running under GNU/Linux. The first of these small processors to be employed for bee-related monitoring and sensing was introduced at the Vermont Hive Monitoring Workshop. Other recently available small processors that are being used in hive monitoring and sensing equipment include: Arduino^®^, Make^®^, Sparkfun^®^ (Niwot, CO, USA), and Adafruit^®^ (New York, NY, USA).

**Figure 8:**
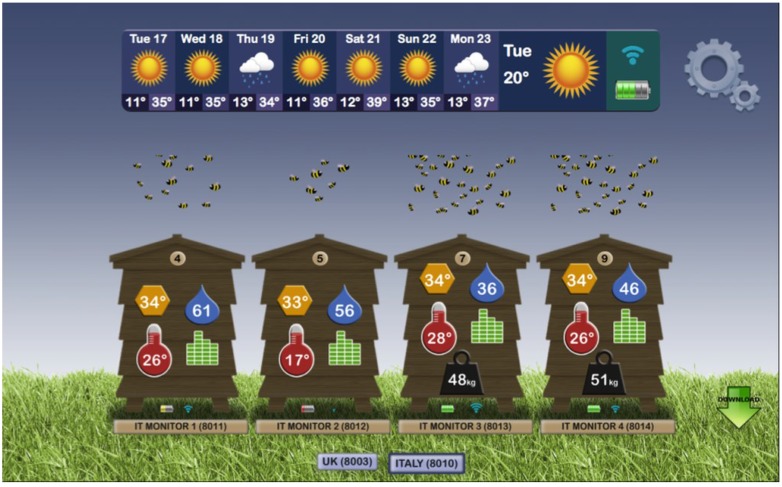
Arnia’s Remote Hive Monitoring web-based reporting system [89]. The interface is intuitive and works well for beekeepers.

Arnia [89], based in the UK, provides individual hive-monitoring systems with cellular communications that are comparable to our pallet-based systems with satellite communications. Founded in 2009, Arnia currently sells their systems in 14 countries. Arnia’s hive systems are distinguished by ready access to hive data via any Internet-enabled device in any web browser (Figure 8) for relatively low-cost, hive-based data acquisition from individual hives.

Arnia’s focus is on backyard beekeepers, students, and other small-scale or networked research projects. Arnia’s systems offer small size, light weight, several sensor options, and established web-based reporting [89]. Their systems are much more mature than most of those offered by more recent entries into the field. Arnia recently supported an experiment involving 75 colonies and reported to us that their graphic user interface worked well, easily and intuitively monitoring different sensors from different hives at different sites. They concluded that it should serve commercial beekeepers with up to eight hives per site, but agree with us that there may be a need for a different approach to very large-scale beekeeping operations or large-scale studies comparing data from hundreds to thousands of hives.

Large commercial beekeeping operations, intensive research projects, and critical applications such as those of the military may require more than simple hive sensor information. Ruggedness, reliability, low maintenance, solar power, and satellite communications are critical issues. These users need an overall management system that combines hive-based measurements, such as from an Arnia kit, our own pallet-based SmartHive^℗^ systems, or those of other suppliers, integrated with technologies such as acoustic scanning for pests, diseases, and pesticide exposure warnings, with infrared scanning to assess colony size and condition and bar codes or RFID tags for asset tracking and inventory control to help them with everything that they do. All of these issues, plus multi-variate data analysis, data visualizations, and advanced data processing tools such as Artificial Neural Networks (ANNs) that can recognize patterns in large data sets, are areas of expertise that our team has been addressing since 1995.

The 50 colonies that we placed online in 1995 and ran for five years quickly demonstrated to us a pressing need for data integration and display. We also learned which sensors were most useful, which were disruptive to bees, and which were problematic in terms of long-term performance. We have 20 years of practical, field-based experience working with electronics and distance communications systems for the monitoring of bees and bee colonies. Arnia has six years of similar research and development and implementation experience. Together we represent nearly 26 years of accumulated field experience with hive monitoring.

### 3.5. Scale Hives and Smart Hives for Bee Management and Monitoring

Beekeeping equipment companies, as well as some scale-manufacturing firms, now sell Scale Hives. Several companies brought their scale hives or presented data from scale hives at the Montana Hive and Bee Monitoring workshop [47,91,92]. Established companies tended to use their own custom data logging systems. The Do It Yourself (DIY) attendees favored Arduino.

The majority of the technologies at the 2nd International Hive and Bee Monitoring Workshop brought scales for one or two hives placed on a hive-scale platform, with a single company offering four or six hive capacity. A few scale hive suppliers can now add other sensors to their scale hives, and a few offer communications, most using wireless or cellular systems. Some of the advantages and limitations of continuous hive monitoring were summarized at the Workshop and in a recently published article [93]. Primarily focused on a review of academic studies, the article did not address some of the most recent developments in technology. More and more companies have started introducing commercial versions of their technologies into the marketplace, and some of these investigators and companies only “publish” their results in their patents received or pending.

A recent monograph, “In Silico Bees”, edited by Devillers [60], provides a broader treatment of hive-based monitoring and sensors. There are chapters covering the use of the information obtained in exploring bee society organization, examining bee decision-making, addressing recruitment and allocation of honey bee foragers, modeling infectious diseases, viewing honey bee ecology from a landscape perspective, addressing Quantitative Structure Activity Relationships (QSAR) modeling of pesticide toxicity to bees with mathematical models for addressing chemical contamination into the hive, as well as agent-based modeling of individual insecticides and models for non-apis bees. As with the book he edited in 2002 on estimating the environmental impact of chemicals to honey bees [37], this book provides detailed case history studies and marks the advent of a new field of study.

All of these new technologies offer opportunities to gather new types of data, often in continuous and real-time mode. That capability then provides a means of networking data sources for real-time monitoring using distance communications. Where available, cellular networks can provide the required communications. Arnia uses cellular networks in Europe. However, in many agricultural regions of the western US, in mountains and in large, isolated areas such as the outback of Australia, satellite communications are the only viable communications option. Here again, satellite communication has become far more affordable. We have found low-cost service packages that are competitive with cellular communication.

### 3.6. Acoustic Detection of Colony Pests, Diseases, and Chemicals

On a parallel track with our military-sponsored development of Smart Hives, we developed the use of recordings of hive sounds, first for early detection of airborne threats from toxic chemicals under a SBIR (Small Business Innovation Research) contract from the US Army Center for Environmental Health Research, then for detection of the presence of bee-pests such as mite and bee diseases under SBIR awards from the US Department of Agriculture, with additional funding from the Montana Department of Commerce Board of Research. The resultant methods and scanners are described by US and Canadian patents [49,94,95].

**Figure 9:**
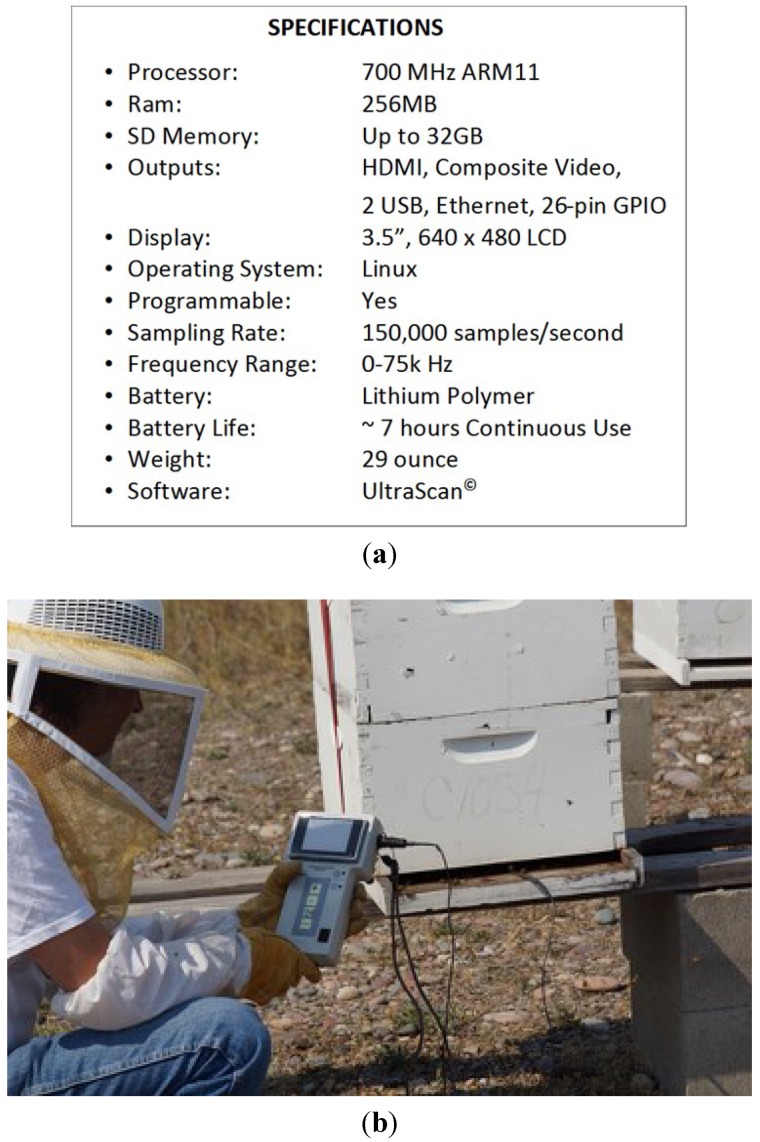
(**a**) Instrument specifications for hand-held acoustic scanner (HAS) for detection of bee pests, diseases, and exposures to harmful chemicals; Bee Alert Technology, Inc., (Missoula, Montana, USA); (**b**) HAS acoustic scanner. A probe microphone is slid into the colony entrance and a 60 s recording taken. The device reads out the detections and probability of detection (e.g., queenless, 95% probability; varroa mites, 28% probability; Africanized bees, 97%, and so forth).

**Figure 10:**
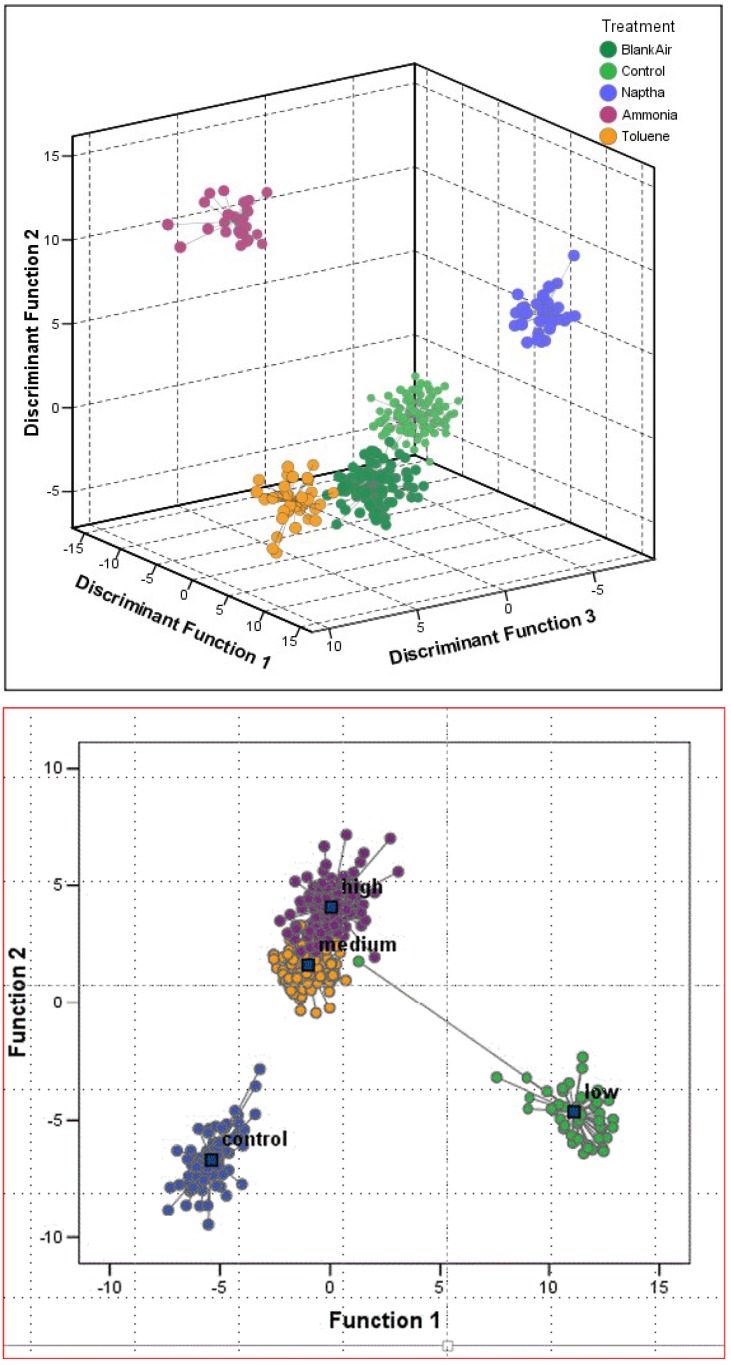
(**a**) Principal Component Analysis (PCA) of honey bee sonograms in response to exposure to a variety of chemicals, published in the patent disclosure [94]; (**b**) PCA of honey bee sonograms relative to infestation rates of varroa mites [95].

Recently, we have just completed another USDA SBIR I project focused on the detection of acute and chronic exposures of bee colonies to pesticides, especially the neonicotinoid pesticides. Preliminary data reveals that while honey bee colonies tend to become noisier when exposed to many of the older organic insecticides, colonies become quieter when exposed to neonicotinoids [96]. Our most significant finding across all of these studies is that not only are the sounds that bee colonies produce altered when the bees are exposed to different stressors, but the colony sounds change in ways which can delineate the type of chemical, pest, and disease (Figure 9a,b and Figure 10a,b) [94,95,96].

**Figure 11:**
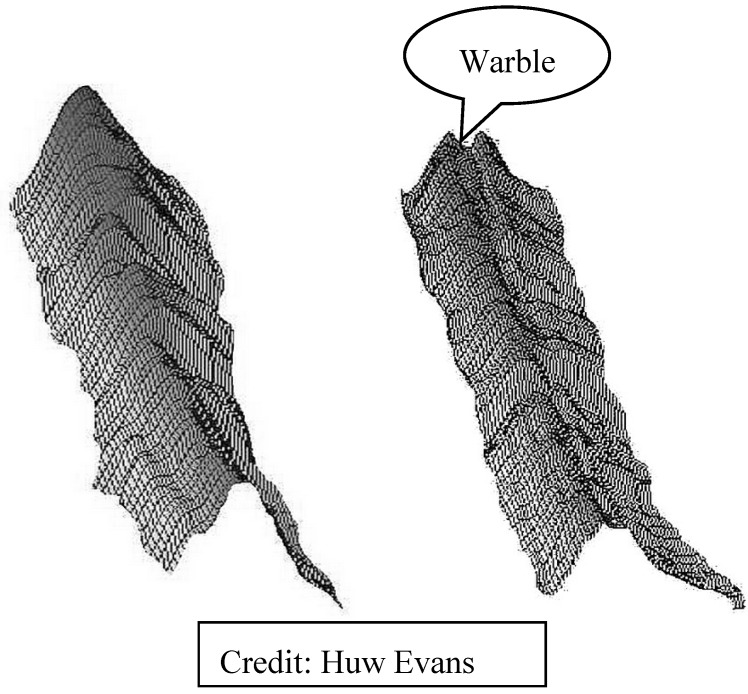
Arnia’s Remote Hive Monitoring. *Normal sonogram* (L.), swarming warble (R) [95].

Other investigators are looking at bee and colony sounds. A research initiative in the UK is currently funding acoustic research. Arnia uses hive sounds in their hive electronics as a generalized indicator of hive activity and for detection of swarming [97,98], as evidenced by a warble in the sonogram of a bee colony (Figure 11). In the US, Trenton Brundage recently patented [99] an external acoustic sensor to monitor bee flight activity.

### 3.7. InfraRed Imaging

**Figure 12:**
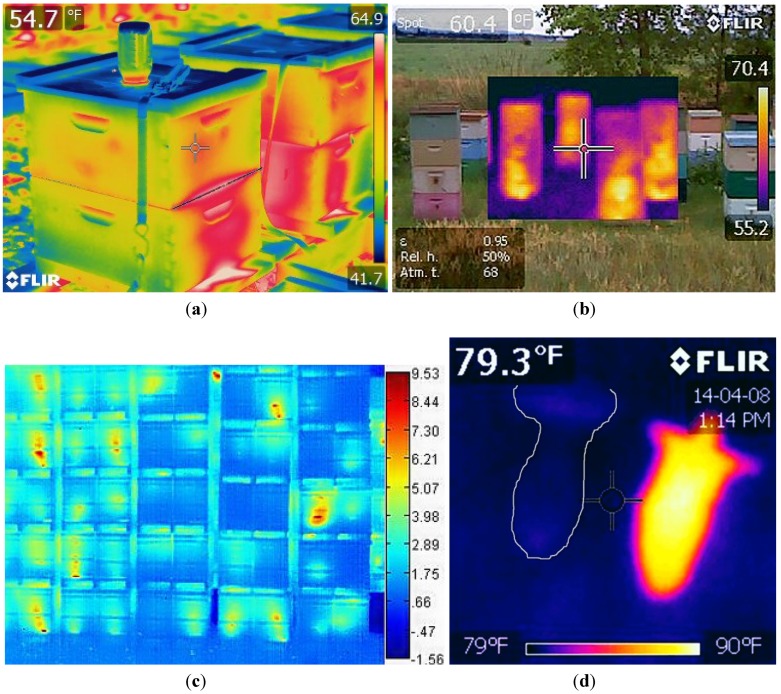
(**a**) Extreme high resolution (307,200 radiometric pixels) image of two bee colonies with feeder on nearest hive; (**b**) Picture in picture, high resolution, (76,800 radiometric pixels); MSX technology fuses infrared and visible light images; (**c**) Moderate resolution (19,200 radiometric pixels); thermal image only of pallets of bee hives stacked in a wintering shed; (**d**) Moderate resolution (19,200 radiometric pixels) image of two queen cells removed from an incubator and left at room temperature for five minutes. Cell on left has stillborn queen; cell on right has live queen.

All objects and animals emit InfraRed (IR) radiation as a function of temperature. Kastberger and Stachel [100] (2003) provided a technical overview of IR measuring systems used with biological systems. They address, among other applications, the imaging of bees and wasps. In 2011, we and our collaborators from Montana State University (MSU) published an article on infrared imaging for non-invasive population assessments of honey bee colony populations in beehives [101]. Since then, we have been investigating other uses of IR cameras for bee management and experimenting with IR cameras of differing resolution capabilities (Figure 12a–d).

Currently, we are working on an IR project for Project *Apis m*, a non-profit foundation that supports bee-related research, and for Blue Diamond Almond growers. We are investigating the efficacy of using IR technology to assess honey bee colony strength. IR imaging offers the possibility of reducing the labor needed to grade colonies by enabling rapid identification of colonies which are weak or borderline. It can also reduce the number of colonies that need to be visually inspected. So far we have focused IR on the bee colonies and on the bees themselves, but there is potential for also using IR to examine chemical signals. Others have used IR to look at the heat profiles of brood nests and heater bees [102,103]. The use of IR for chemical vapor detection was documented in 1990 by Carr *et al.* [104]. Their paper describes the test and analysis of spectrally modified thermal-imaging systems designed to detect chemical vapor clouds.

## 4. Agricultural and Research Applications

### 4.1. Improving Apiary Management

Technologies described herein provide an overview of modern tools which can be used individually and in combinations for better bee management and for innovative research regarding bees, pollinators, crops, and agro-ecosystems. Many of these methods and devices came about because the US military wanted rapid communication, especially if bees were to be used for surveillance to protect against releases of chemical and biological warfare agents. These same technologies can now provide early warnings of pesticide incidents to beekeepers and possibly warn of the arrival of new pests and diseases in bees, crops, and orchards. For large-scale commercial beekeepers who may manage thousands or tens of thousands of colonies, often located over large areas, sometimes even on opposite coasts, there are now options for monitoring colony status in remote areas without having to send a crew to check them. Others of these new tools may reduce or eliminate the need to open colonies to visually inspect them or, at least, to identify colonies that need attention.

Precision agriculture is already changing the science, environmental practices, and economics of livestock and crop management, and concurrently of crop science, environmental practices, and production economics. The latter is especially important to producers of agricultural commodities because it can boost their competitiveness through more efficient practices by reducing costs and, hopefully, increasing yields.

The honey bee industry has not had anything similar to the RFIDS used with cattle or GPS/Satellite yield mapping of crops. Similarly, bee and pollinator science has been held back, especially for field studies, by a lack of real-time quantitative data acquisition systems. Publication of the honey bee genome [105] was a milestone in understanding bees at a molecular level. We anticipate that hive and bee monitoring technologies may have a similar transformative effect on bees, pollination, and crops.

New bee-centric technologies for field use, and, equally important, the transfer of these technologies from research and military applications into affordable, reliable, commercially available devices for use by researchers, beekeepers, and growers are essential given current concerns about worldwide declines of pollinators. Better data acquisition with sophisticated analysis, modeling, and visualization programs offers significant potential for rapidly improving bee management and bee science, especially with respect to issues such as pollinator efficacy, seed production, and plant security.

Obviously, a beekeeper with apiaries hundreds of kilometers away can save a considerable amount of money by having a report delivered to a cell phone, saving the cost of salaries, employee benefits such as workman’s compensation insurance, fuel, meals, and lodging expenses of sending a crew to check the distant colonies. For that purpose, a Scale Hive and some basic weather information can be invaluable.

In trying to detect a pesticide exposure incident, hive weight is a relatively insensitive factor, whereas a bi-directional bee counter can readily identify an event and provide accurate, time-related data concerning the time, date, and degree of loss by reporting the number of bees leaving the hive and how many returned. Our data from 50 colonies over several years indicates that the return rate for a healthy hive is in the mid-to-high 90th percentile. Acute exposure to a poison may drop this to 80% or lower within a day. A chronic exposure event is more likely to play out over a series of days, with the colony losing another 1%–5% of the bees expected to return each day. If successful, our acoustic system may be able to provide comparable information for far less cost with a simpler system than a hive-entrance counter. Microphones are relatively inexpensive compared to counter devices.

Our IR imaging of colonies was initiated so that a soldier who is unfamiliar with honey bee life histories could determine whether a sentinel colony was alive, of suitable population size, queen-right, and in good condition without knowing much about beekeeping and colony inspections. Similarly, growers who rent colonies for pollination have to trust their beekeeper. Some pay a premium for colonies of a certain size. Growers often hire bee brokers and grading crews, who score the condition and size of a percentage of 5%–15% of the colonies being rented.

If we can successfully calibrate and verify that an IR camera can score hives with equal or better accuracy than opening the hive and visually inspecting a colony, any grower could verify the condition of colonies delivered without opening hives or knowing how to manipulate bees. In addition, it should be possible to score/grade every colony, not just a subset of the colonies offered for rent. Considering that the almond growers rent more than 1.6 million colonies each year and that 2015 rental fees were in the $ 140 to $ 170 range or higher per colony, it is important that the grower gets fair value for their investment.

The beekeepers also gain advantages with use of an IR camera. Colonies are shipped from as far as the east coast to the west coast, with many being shipped in the fall or winter from cold climates where it is difficult, if not impossible, to inspect bees at the point of origin. Weak and dead colonies are often discovered in California after delivery when the hives are opened for feeding and inspection. Shipping a substandard or dead bee colony and its hive to California and returning it without being able to rent it is a major cost, since freight can run to $ 90 or more per colony each way.

Small-scale backyard beekeepers may take an interest in these technologies, but inspecting one or two colonies in their backyard using these technologies would not be cost-effective. Although not necessarily needed for good bee management of a few colonies, some of these technologies may be warranted from the perspective of learning about bees or just the fun of trying something different. In Ireland a group of beekeepers bought eight of Arnia’s hive monitoring systems and they are working with students from a local school to conduct simple experiments and learn more about bees.

### 4.2. Pollinator Research

At the opposite end of the spectrum are basic science studies using these new methodologies. For example, Palmer, USDA-ARS, Iowa, and his team looked at the sugar content of nectar, mass spectrometry chromatography of volatile compounds, and PERs assays [106] to examine new varieties of perennial soybeans. Only a few proved to be attractive to honey bees. 

Debnam, one of our graduate students at UM, is conducting his thesis research on determining what effect, if any, native plant diversity may have on honey bee foraging at the landscape scale. Individual bees are not tracked but rather the foraging activity of the honey bees within an entire area is mapped using LIDAR. To accomplish this, 154 one-meter plots were planted with plants native to the Rocky Mountain Region of Montana. Each plot contained 20 individual plants but varied in diversity between 1 and 5 plant species. In May of 2014, all the plant species within the plots were in bloom and LIDAR scans were run from 2 p.m. to 4 p.m. three times a week.

**Figure 13:**
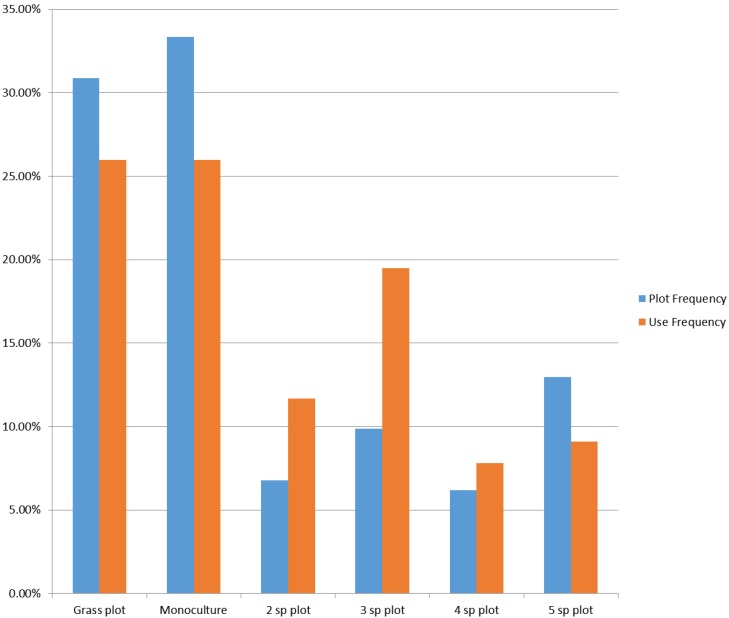
Honey bee use of plots of varying plant diversity as mapped by use of bee-mapping LIDAR.

Honey bees that foraged within the diversity plot area preferred the diverse plots over the monoculture plots (Chi square *P* = 0.016). Forty percent of the bees that foraged within the diversity plots were mapped over an area that encompassed only 23% of the diversity plot. Within this 23% plot area that the honey bees elected to forage, the bees were most frequently found within the areas that contained the three species plots, suggesting that the bees preferred the plots with three-species diversity (Figure 13). Similar studies have been conducted, but most involved groups of students standing in a field with click counters. The downsides of this manual approach are: (1) the presence of observers in the field is likely to alter bee foraging; and (2) observer bias that causes each observer to have different ability to focus on the task, to see, and to count rapidly moving small insects. LIDAR removes all of these variables and provides decimeter locations of each observed and counted bee.

Working with researchers and seed producers in the US and overseas we have been able to improve seed set and pollination efficacy by conditioning bees to the crop of interest. This is especially useful for crops that bees are not naturally attracted to, such as onions. For countries which have limited numbers of bee colonies for pollination, and who do not wish to import bees from other areas due to concerns about introducing new bee diseases and pests, the ability to improve pollinator fidelity and to increase the numbers of bees on blooms using locally available bees is a preferred option. Though we know that others working for DARPA have managed to condition moths and wasps to respond to scents in a laboratory or tent, we have never seen either of these insect groups entraining at our conditioners in the field. Wasps and moths sometimes discover the conditioning trays, but they never discover the “target” of interest. However, we know that we can successfully condition both honey bees and bumble bees for scent-mediated search.

### 4.3. Passive and Active Detection of Exotic Species and Diseased Plants

**Figure 14:**
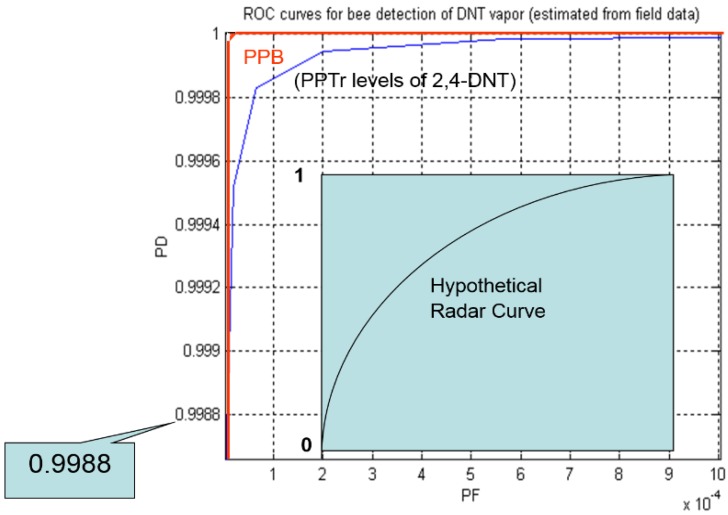
Receive Operating Characteristic curve (ROC) for bee detection of 2,4-DNT in soil, derived from DARPA funded trials, based on a figure we provided to a Rand report on Alternatives to Landmine Detection, 2003 [12].

Our military trials verified bee detection of non-floral and non-pheromone chemicals at parts per billion (PPB)/parts per trillion (PPT) levels (Figure 14), chemicals one might not expect bees to be able to detect at all [12]. We have videos of bees locking onto an airborne vapor trail while still tens of meters away from the vapor odor source, measured at just a scant few PPT. When bees cross a vapor trail of interest to them, they turn toward the source, following the scent upwind. This detection of a scent meters away from a low (below 15 PPT) concentration source implies instantaneous detection by bees at levels even lower, nominally parts per quadrillion (PPQ).

This detection behavior by bees following scents and chemical signals known to be attractive to them is passive behavior. However, it also can be used actively, by conditioning them to seek out scents of interest, whether free-flying bees in the field [70,71,72] or constrained in a laboratory using PERs [17,18,31,107]. Other investigators use honey bees and bumble bees to carry or vector microbial biocontrol products to control crop diseases and insect pests of crops [108,109,110,111,112]. Peter Kevan at the University of Guelph calls this “bee-vectoring biocontrol”. He relies on bees to passively deliver biocontrol microbes to specific crops. For example, his team uses *Clonostachys rosea* to control *Sclerotinia sclerotiorum*, which causes sunflower head rot, and *Beauveria bassiana* to control banded sunflower moth [108,109,110,111]. Recent trials reduced severity by 70%–100% and increased sunflower yield by 20% [112] or more.

Similarly, DARPA has funded research on a variety of scenarios whereby larger insects like cockroaches and moths are employed to carry chemical reactive or electronic sensors [40]. Size, weight requirements for battery power, and information retrieval present logistical challenges with smaller insects such as bees.

A related and intriguing question that we are studying is whether bees can be used for finding introduced plants and animal species in native habitats. Our collaborators in New Zealand have investigated using bees to detect human diseases (Suckling and Sagar [107]) and verified that bees could detect the scent of *Mycobacterium tuberculosis*. We have just now begun to explore whether bees can serve similar functions for detecting infected plants and other biosecurity targets.

## 5. Resolving Questions about Dance Language and Scent Detection

Now that we have covered bees as biosensors and contemporary biosensor technologies, we can return to the premise posed in our introduction: that *scent-directed search is under-valued by the focus on a dance language*. Harmonic radar technology and, we suspect, bee mapping LIDAR, have finally provided technologies for answering long-standing questions about the honey bee dance language.

By 2004, Reinhard had concluded that floral scents induce recall of visual and navigational memories in honey bees [113]. Then, in 2005 and 2007 [56,114], Riley and his team reported that the dance communication/flight navigation system of the honey bee was used by forager bees to get them to the vicinity of a food source, but not to the specific location. Only 2 out of 19 tracked bees located the feeder that the dance communication indicated. The remaining bees initiated local searching maneuvers that ranged as far as 200 m from the feeder and lasted as long as 20 min. Other recent researchers using harmonic radar had similar difficulty in showing the dance communication/flight navigation system’s ability to direct foragers to unscented food sources [115].

The dance communication/flight navigation system of the honey bee is not precise. At best it only provides general distance and direction information. The language appears to be only as helpful as directing a person to an office building, but not informing that person where in the building to go. To discover the location of the appropriate office, the person would need to rely on other information such as a building directory. For the honey bee that information would be the scent she gleaned from the dancing forager, her own individual foraging experience, and presumably both scent and visual clues that she uses near the location of the food source.

Despite historical anecdotes that bees have a poor sense of smell, using PERS, video imaging of free-flying bees, and LIDAR, we have learned much about bee foraging and have published in Technical Reports to the US military Receiver Operating Characteristic curves (ROCs) for honey bee detection of chemicals that one might not expect them to be able to discern. The term Receiver Operating Characteristic came from World War II radar operators who tested the ability to determine whether a blip on the screen represented a signal (object) or noise.

Our ROCs for scent detection by free-flying bees were independently verified by US Army scientists, based on extensive video documentation, and by Sandia scientists who provided the chemical concentrations in the air just above buried substances. Published by the Rand Corporation [12], the ROCs for bee detection of 2–4 dinitrotoluene (DNT) in soils at PPB and at PPT levels were nearly perfect (Figure 14). A perfect sensor would have a value of 1.0 for the area under the curve (AUC), a theoretically perfect test (appearing in the upper left hand corner of chart). An AUC of 0.5 indicates no discriminative value. Bees at PPB and PPT scent concentrations scored above 99.9% for instantaneous scent detection.

## 6. Summary and Conclusions

This article details major steps in the development of using bees as biosensors, as carriers of biosensors, and even as delivery vectors for sensors and biocontrol agents to plants and other targets. A wealth of new technologies and tools have appeared over the past decade, and the number and rate at which these are appearing is accelerating. How all of these technologies are going to be used to detect and decipher chemical signals between organisms, whether with other bees, other species of insects, between bees and plants, and even more unusual combinations such as bees and humans or even livestock, remains to be seen. Clearly, we know that the enabling technologies will require an appropriate development of analytical software; the data flows from free-flying biosensors can rapidly generate enormous amounts of information. Data mining and interpretation will present new challenges. Twenty years ago we learned that putting 50 colonies of bees in hives equipped with multiple sensors and weather stations yielded the data equivalent of going from a drip at the end of a hose to the full blast of a fire hose. Others have now recognized and begun to discuss this issue and other challenges in the development of precision agriculture for beekeeping [116].

We also discovered that trying to guess which sensors provided the most useful information, which were difficult to maintain in a hive environment, and which were perceived, possibly harmed, or were attacked by bees is not possible without experimentation and rigorous scientific analysis of the results. Others who are now using these or similar technologies, as evidenced by the presentations at the 2nd International Workshop on Bee and Hive monitoring, have begun the same path of discovery [47,91]. Enthusiasm was high, although some predications and assessments will likely change based on more than a season or two of data.

Finally we recommend caution about sweeping judgments in preliminary stages of use and equally important, experience with emerging systems—the test of time has a maddening way of over-turning preliminary results and assumptions. Just having the technology is exciting and opens up a wealth of opportunities and new perspectives with respect to agro-ecosystems and production agricultural commodities.

### Research Initiatives

Currently, a plethora of emergent honey bee monitoring and biosensor technologies are poised to enter the market place at affordable prices. We have attempted to provide a thorough survey, emphasizing the diversity of these innovations and their historical context, so that the many innovators who have worked tirelessly to provide them are not forgotten. It is not surprising then that an anonymous reviewer of this manuscript would ask specifically for more information about LIDAR studies and more general questions about specific projects and/or programs that could be undertaken to achieve specific objectives in the near term.

With respect to LIDAR, the technology was developed for defense applications by the US Army. As such, strict restrictions are imposed regarding the export of methods, equipment, information, and uses. No other work is referenced for this method because only two fully operational bee mapping instruments exist in the world. The development of these instruments involved over a decade of closely audited defense research. Access to this information is made to appropriate parties on a case by case basis. The information cannot, unfortunately, be published in an open literature article.

However, bee mapping LIDAR has dual usage. The instruments can be used and studies reported for non-military applications such as research and agriculture. We summarized herein new initiatives and provided a few preliminary findings. We look forward to exploring other collaborative uses of these LIDAR systems with other investigators.

General initiatives suggested by the reviewer included studies monitoring individually marked bees to visualize and analyze movement patterns of free-flying bees, decoding the waggle dance, exploring hive status (population size, health, dynamics), using chemical sensory abilities of both constrained and free-flying bees, and many more research opportunities. The reviewer emphasized that for each a primary objective should be to clearly identify the applications (research, biosecurity, Eco monitoring, beekeeping), the benefits, and drawbacks. We are thankful to the reviewer for these comments and agree. Presently, for many of the recommended initiatives, current information is too limited to permit a thorough analysis for benchmarking these new technologies. Where it is available, we have cited appropriate review articles that discuss these issues in more depth. We believe that there is much scope for future research in this area.
